# Plasma N-Cleaved Galectin-9 Is a Surrogate Marker for Determining the Severity of COVID-19 and Monitoring the Therapeutic Effects of Tocilizumab

**DOI:** 10.3390/ijms24043591

**Published:** 2023-02-10

**Authors:** Hiroko Iwasaki-Hozumi, Yosuke Maeda, Toshiro Niki, Haorile Chagan-Yasutan, Gaowa Bai, Takashi Matsuba, Daisuke Furushima, Yugo Ashino, Toshio Hattori

**Affiliations:** 1 Research Institute of Health and Welfare, Kibi International University, Takahashi 716-8508, Japan; 2 Viral Section, Department of Microbiology, Faculty of Life Sciences, Kumamoto University, Kumamoto 860-8556, Japan; 3 Department of Immunology, Faculty of Medicine, Kagawa University, Kagawa 761-0793, Japan; 4 Department of Animal Pharmaceutical Science, School of Pharmaceutical Science, Kyusyu University of Health and Welfare, Nobeoka 882-8508, Japan; 5 Faculty of Medicine School of Health Science, Kagoshima University, Kagoshima 890-8544, Japan; 6 Department of Respiratory Medicine, Sendai City Hospital, Sendai 982-8502, Japan

**Keywords:** coronavirus disease 2019, N-cleaved-Gal9, surrogate marker, proteolysis, severity, therapeutic effects, tocilizumab, soluble interleukin-2 receptor, matrix metalloprotease-9, C-reactive protein

## Abstract

Galectin-9 (Gal-9) is known to contribute to antiviral responses in coronavirus disease 2019 (COVID-19). Increased circulating Gal-9 in COVID-19 is associated with COVID-19 severity. In a while, the linker-peptide of Gal-9 is susceptible to proteolysis that can cause the change or loss of Gal-9 activity. Here, we measured plasma levels of N-cleaved-Gal9, which is Gal9 carbohydrate-recognition domain at the N-terminus (NCRD) with attached truncated linker peptide that differs in length depending on the type of proteases, in COVID-19. We also investigated the time course of plasma N-cleaved-Gal9 levels in severe COVID-19 treated with tocilizumab (TCZ). As a result, we observed an increase in plasma N-cleaved-Gal9 levels in COVID-19 and its higher levels in COVID-19 with pneumonia compared to the mild cases (healthy: 326.1 pg/mL, mild: 698.0 pg/mL, and with pneumonia: 1570 pg/mL). N-cleaved-Gal9 levels were associated with lymphocyte counts, C-reactive protein (CRP), soluble interleukin-2 receptor (sIL-2R), D-dimer, and ferritin levels, and ratio of percutaneous oxygen saturation to fraction of inspiratory oxygen (S/F ratio) in COVID-19 with pneumonia and discriminated different severity groups with high accuracy (area under the curve (AUC): 0.9076). Both N-cleaved-Gal9 and sIL-2R levels were associated with plasma matrix metalloprotease (MMP)-9 levels in COVID-19 with pneumonia. Furthermore, a decrease in N-cleaved-Gal9 levels was associated with a decrease of sIL-2R levels during TCZ treatment. N-cleaved-Gal9 levels showed a moderate accuracy (AUC: 0.8438) for discriminating the period before TCZ from the recovery phase. These data illustrate that plasma N-cleaved-Gal9 is a potential surrogate marker for assessing COVID-19 severity and the therapeutic effects of TCZ.

## 1. Introduction

Coronavirus disease 2019 (COVID-19), first reported in Wuhan, China, is caused by severe acute respiratory syndrome coronavirus 2 (SARS-CoV-2). As of 18 August 2022, over 590 million cases have been confirmed globally as COVID-19, with more than 6.4 million deaths [1]. Among symptomatic COVID-19 cases, most patients developed mild (40%) or moderate (40%) diseases, while the remaining 20% developed severe or critical diseases requiring oxygen support with complications including respiratory failure, acute respiratory distress syndrome (ARDS), sepsis, thromboembolism, and multiorgan failure. Extensive scientific research has contributed to developing therapies for COVID-19. Despite the knowledge gained from various analyses, its pathophysiology must still be better understood to control the complexity of immune responses to COVID-19.

Well-functioning immune responses inhibit virus replication without tissue injury with no or mild symptoms. In contrast, excessive immune responses are known to cause local and systemic hyperinflammation with tissue damage leading to severe COVID-19 [2]. The severity of COVID-19 is associated with the increase in circulating proinflammatory mediators, including interleukin (IL)-6, soluble IL-2 receptor (sIL-2R), C-reactive protein (CRP), and ferritin [3,4,5,6]. Thus, one aspect of COVID-19 pathophysiology is characterized as a cytokine storm. Paradoxically, the opposing mechanisms inducing high morbidity and mortality in COVID-19 are immunosuppression which causes a collapse in the host’s protective response, leading to viral dissemination and organ injury [7]. Intriguingly, the cytokine storm is believed to occur at the late stage of severe and critical COVID-19, while immune responses are suppressed in the early stage [8].

Galectin-9 (Gal-9) is a β-galactoside-binding lectin expressed in various cell and tissue types associated with the immune system both intra- and extra-cellularly. Gal-9 structurally consists of two homologous carbohydrate-recognition domains at the N- and C-terminus (NCRD and CCRD, respectively) linked by a linker peptide that is highly susceptible to proteolysis [9,10,11]. The proteolysis separates NCRD and CCRD with the attached truncated linker peptide that differs in length depending on the type of proteases (here defined as N- and C-cleaved-Gal9, respectively), and is supposed to erase the activities of Gal-9, whereas there are several studies suggesting that proteolytic products of Gal-9 exert different bioactivities [11,12]. Gal-9 is a molecule that positively and negatively adjusts immune systems as both a contributing factor in cytokine storm and an immunosuppressive molecule inducing, for instance, T-cell exhaustion and apoptosis [9,13,14,15,16]. According to many previous studies, the circulating Gal-9 levels fluctuate in various diseases, including immunological diseases and infectious diseases [9,17,18].

**Figure 1:**
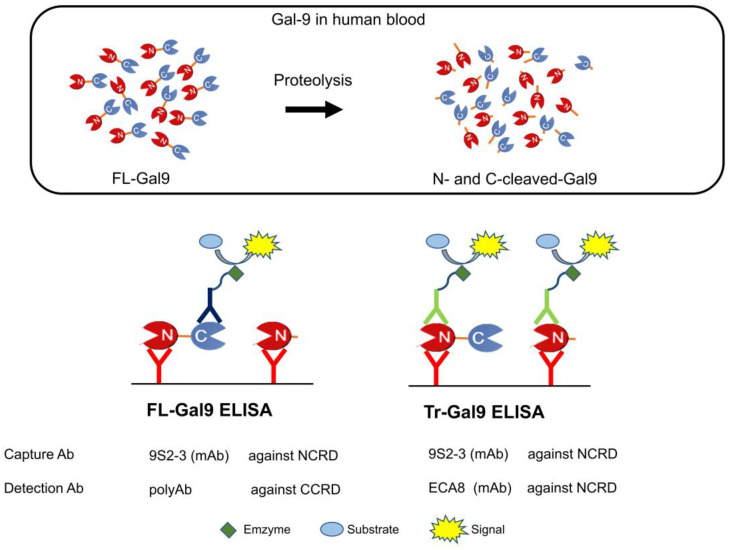
Schema showing the detection systems in two types of Gal-9 ELISA. Full-length Gal-9 (FL-Gal9), and NCRD and CCRD with attached truncated linker peptides that differ in length depending on the type of proteases (N-cleaved-Gal9 and C-cleaved-Gal9, respectively) are present in human blood. The concentration of FL-Gal9 was measured using FL-Gal9 ELISA. The concentration of Tr-Gal9, a mixture of FL-Gal9 and N-cleaved-Gal9, was measured using Tr-Gal9 ELISA. The capture and detection antibodies and detected fragments in each ELISA are shown. The concentration of N-cleaved-Gal9 was calculated using both ELISA by subtracting the concentration measured by FL-Gal9 ELISA from that measured by Tr-Gal9 ELISA. Gal-9: galectin-9, ELISA: enzyme-linked immunosorbent assay, CRD: carbohydrate-recognition domain, NCRD: N-terminal CRD, CCRD: C-terminal CRD, mAb: monoclonal antibody, polyAb: polyclonal antibody.

The concentrations of circulating Gal-9 are reported to significantly increase in COVID-19, and they reflect the severity of the disease [15,19,20,21,22]. Each of these results is based on a different measurement method, and there are no mentions of what type of Gal-9, either intact or degraded, was measured in the studies. We have accurately investigated the relevance of plasma Gal-9 levels to COVID-19 severity using two types of our originally developed enzyme-linked immunosorbent assay (ELISA) [23]. Most of the Gal-9 activities reported so far are exhibited by intact Gal-9 that is full-length (FL) Gal-9. To measure intact Gal-9, we have developed FL-Gal9 ELISA combining the capture and detection antibodies that recognize NCRD and CCRD, respectively (Figure 1). Moreover, using the capture and detection antibodies both against NCRD, we have developed Tr-Gal9 ELISA that enabled us to measure both Gal-9 containing NCRD (FL-Gal9 and N-cleaved-Gal9) (Figure 1). Using both ELISA, we have measured plasma FL-Gal9 and Tr-Gal9 levels in severe cases associated with pneumonia and moderate cases with mild symptoms of COVID-19. As a result, both FL-Gal9 and Tr-Gal9 levels were significantly higher in COVID-19 with pneumonia, whereas Tr-Gal9 levels showed a higher accuracy for discriminating the two groups compared to FL-Gal9. Tr-Gal9 but not FL-Gal9 levels were associated with inflammation, coagulopathy, and respiratory disorder levels. We have also measured the time course of FL-Gal9 and Tr-Gal9 levels in severe and critical COVID-19 treated with tocilizumab (TCZ), a monoclonal antibody against both membrane-bound and soluble IL-6 receptors [24,25,26]. As a result, Tr-Gal9 levels markedly decreased more than FL-Gal9 levels after TCZ administration.

In the disease progression of COVID-19, Gal-9 has the potential to be cleaved by proteases produced in inflamed tissues. Therefore, cleaved Gal-9 intermediates that are stable to a certain degree may be a more effective marker for the diagnosis of COVID-19 than active but fragile FL-Gal9. Proteases including matrix metalloproteases (MMPs), which are considered to cleave Gal-9, are involved in the aggravation of COVID-19 [27,28,29,30]. The cleaved forms of Gal-9 are the products of a complex process of Gal-9 production and its degradation; therefore, the measurement of cleaved Gal-9 may be more effective for assessing the pathophysiology and severity of COVID-19.

In this study, we determined plasma levels of FL-Gal9, Tr-Gal9 and N-cleaved-Gal9 to compare their diagnostic potentials in COVID-19 with mild and severe cases and evaluated the associations of their levels with therapeutic effects of TCZ. N-cleaved-Gal9 levels was calculated by subtracting FL-Gal9 levels from Tr-Gal9 levels. In addition, plasma levels of MMP-2 and MMP-9, two candidate proteases to cleave the linker peptide of Gal-9 [9], were determined to examine their association with the N-cleaved-Gal9. We aimed to clarify the potential of N-cleaved-Gal9 as a marker used for the management of COVID-19 patients.

## 2. Results

### 2.1. Characteristics of CV, CP, and ID Patients Enrolled in the Study

**Figure 2:**
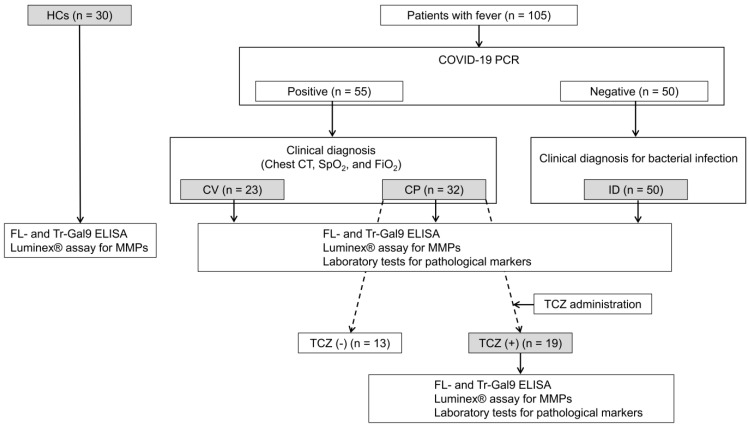
Flow chart of the study. The gray boxes show the subjects investigated in the study. HCs: healthy controls, COVID-19: coronavirus disease 2019, CV: COVID-19 patients with mild symptoms, CP: COVID-19 patients associated with pneumonia, ID: patients suspected with bacterial infections, TCZ (+): CP patients treated with tocilizumab (TCZ), with severe symptoms being managed in an intensive care unit (ICU), TCZ (−): the other CP patients not treated with TCZ, chest CT: chest-computed tomography, SpO2: percutaneous oxygen saturation, FiO2: fraction of inspiratory oxygen, MMP: matrix metalloproteinase.

A total of 105 patients with fever were enrolled in the study, of which 55 were diagnosed with COVID-19 and 50 were suspected of bacterial infections (ID) (Figure 2). COVID-19 patients comprised 23 patients with mild symptoms (CV) and 32 patients associated with pneumonia (CP). The basic characteristics of CV, CP, and ID patients are summarized in Table 1. The median ages of CV, CP, and ID were 22 (range: 19–102), 53 (range: 20–99), and 80 (range: 23–94), respectively. CV, CP, and ID showed normal levels of platelet (PLT) and red blood cell (RBC) counts, except for the aberrant value of white blood cell (WBC) counts in ID. CP patients suffered from various comorbidities, of which hypertension and diabetes mellitus were the most common, while a few CV patients suffered from comorbidities. Most ID patients had cancers, followed by cerebrovascular and cardiovascular diseases.

### 2.2. Levels of Plasma Gal-9, Plasma MMPs, and Specific Pathological Markers in Different Severity Groups of COVID-19

**Figure 3:**
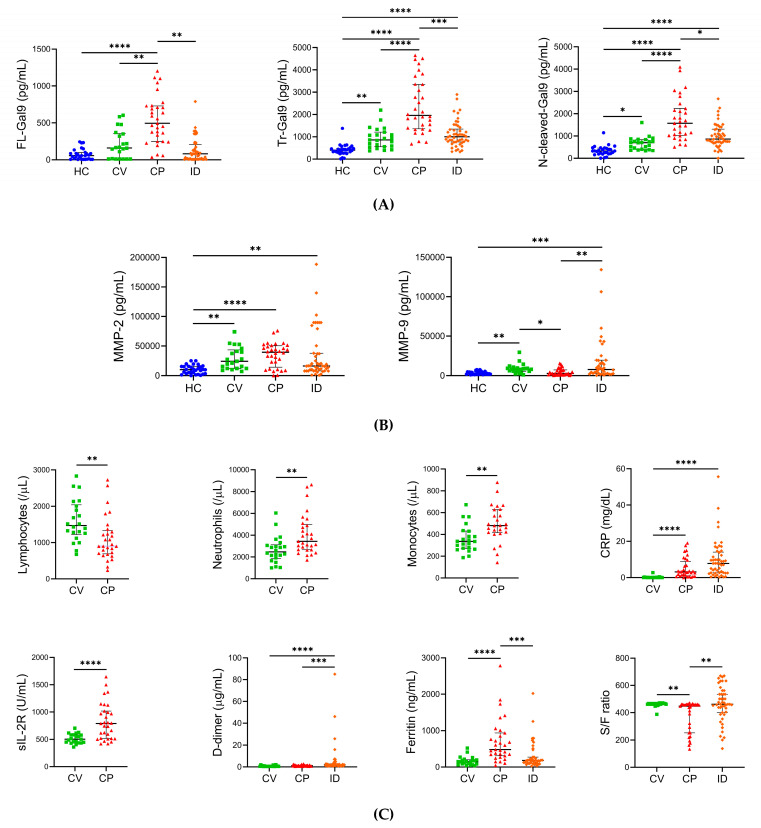
Gal-9, MMPs, and specific pathological marker levels in healthy controls, COVID-19, and bacterial infections (**A**–**C**). (**A**) Plasma FL-Gal9, Tr-Gal9, and N-cleaved-Gal9, (**B**) plasma MMP-2 and MMP-9, and (**C**) specific pathological marker levels in healthy controls, CV, CP, and ID are shown. The median for each parameter is indicated with IQR. CRP: C-reactive protein, sIL-2R: soluble interleukin-2 receptor, S/F ratio: SpO2/FiO2 ratio, * *p* < 0.05, ** *p* < 0.01, *** *p* < 0.001, **** *p* < 0.0001.

We determined the baseline of plasma Gal-9, MMP-2, and MMP-9 levels and the specific pathological marker levels in healthy controls, CV, CP, and ID at the initial visits. Both FL-Gal9 (495.0 pg/mL, IQR (interquartile range): 247.8–730.8 pg/mL) and Tr-Gal9 (1961 pg/mL, IQR: 1359–3353 pg/mL) levels in CP were significantly higher compared to those in healthy controls, CV, and ID (Figure 3A). Likewise, N-cleaved-Gal9 levels in CP (1570 pg/mL, IQR: 1021–2232 pg/mL) were significantly higher compared to those in CV (698.0 pg/mL, IQR: 402.3–840.2 pg/mL) and ID (866.0 pg/mL, IQR: 727.2–1309 pg/mL) as well as healthy controls (326.1 pg/mL, IQR: 196.5–459.0 pg/mL). Notably, we found more significant differences in Tr-Gal9 and N-cleaved-Gal9 levels (*p* < 0.0001) between CV and CP compared to those in FL-Gal9 levels (*p* < 0.01).

MMP-2 levels in CV (24,015 pg/mL, IQR: 12,183–43,476 pg/mL), CP (39,472 pg/mL, IQR: 14,351–50,475 pg/mL), and ID (16,416 pg/mL, 8119–37,693 pg/mL) were higher compared to those in healthy controls (10,252 pg/mL, IQR: 3442–15,627 pg/mL) (Figure 3B). Its levels in CP were markedly elevated. On the other hand, MMP-9 levels in CP (2771 pg/mL, IQR: 1423–7321 pg/mL) were significantly lower compared to those in CV (8122 pg/mL, IQR: 5527–9691 pg/mL) and ID (7868 pg/mL, 2643–19,484 pg/mL) and comparable to those in healthy controls (2373 pg/mL, 1735–4983 pg/mL).

CP showed lymphopenia (900.0/μL, IQR: 690.2–1333/μL) as reported in severe COVID-19 [31,32,33], while CV maintained normal levels of lymphocyte counts (Figure 3C). The neutrophil and monocyte counts in CP were significantly higher than those in CV. We observed significantly higher levels of CRP, sIL-2R, and ferritin, which mediate inflammatory conditions, in CP compared to those in CV; thus, inflammation levels were markedly exacerbated in CP more than in CV. There were no significant differences in D-dimer levels between CV (0.5500 μg/mL, IQR: 0.4400–0.9200 μg/mL) and CP (1.135 μg/mL, IQR: 0.6575–1.508 μg/mL), suggesting that the levels of coagulopathy in CP were comparable to those in CV. We also observed significantly lower levels of D-dimer and higher levels of ferritin in CP compared to ID, implying that inflammation was enhanced in severe COVID-19 more than in bacterial infections, while COVID-19 patients did not develop coagulopathy compared to bacterial infections. CP patients’ respiratory functions were significantly exacerbated more than those in CV and ID according to ratio of percutaneous oxygen saturation (SpO2) to fraction of inspiratory oxygen (FiO2) (S/F ratio).

### 2.3. Associations between Plasma FL- and N-Cleaved-Gal-9 in COVID-19 with Pneumonia

We investigated the associations of plasma FL-Gal-9 levels with plasma N-cleaved-Gal9 levels in healthy controls, CV, CP, and ID. Interestingly, we observed a positive correlation between FL-Gal9 levels and N-cleaved-Gal9 levels in CP, whereas no correlations were found between them in healthy controls, CV, and ID (Figure S1). These data suggested that FL-Gal9 could be processed by proteases almost as soon as it was released into the peripheral blood from tissues in CP.

### 2.4. Associations of Plasma N-Cleaved-Gal9 with Plasma MMP-9, and Specific Pathological Markers in COVID-19 with Pneumonia

**Figure 4:**
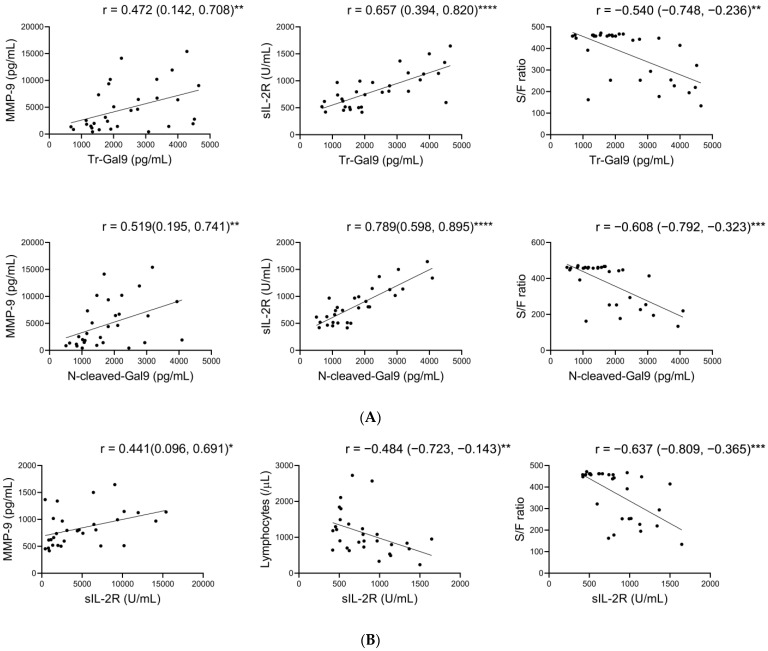
Correlations of Tr-Gal9 and N-cleaved-Gal9 with MMP-9 and specific pathological markers, and those of sIL-2R with MMP-9 and specific pathological markers in COVID-19 with pneumonia (**A**,**B**). Scatter plots show Spearman’s rank correlations of (**A**) plasma Tr-Gal9 and N-cleaved-Gal9 levels with plasma MMP-9 levels, sIL-2R levels, and S/F ratio and (**B**) sIL-2R levels with plasma MMP-9 levels, lymphocyte counts, and S/F ratio in CP. r: correlation coefficient, ( ): 95% confidence interval (CI), * *p* < 0.05, ** *p* < 0.01, *** *p* < 0.001, **** *p* < 0.0001.

Here, we analyzed the associations of plasma FL-Gal9, Tr-Gal9, and N-cleaved-Gal9 levels with plasma MMP-2 and MMP-9 levels and the specific pathological marker levels. In CP, Tr-Gal9 and N-cleaved-Gal9 levels positively correlated with MMP-9, CRP, sIL-2R, D-dimer, and ferritin levels and negatively correlated with lymphocyte counts to a greater degree than FL-Gal9 (Figure 4A, Table S1). Tr-Gal9 and N-cleaved-Gal9 levels also negatively correlated with S/F ratio in CP, whereas there was little correlation between them in CV and ID. Moreover, N-cleaved-Gal9 levels showed higher correlations with MMP-9, CRP, sIL-2R, and D-dimer levels and S/F ratio compared to Tr-Gal9.

sIL-2R is produced from the enzymatic cleavage of IL-2Rα chain on cells such as lymphocytes, where MMP-9 is able to mediate its cleavage [34,35,36]. sIL-2R is processed by MMP-9 in inflamed lung tissues and then released into the peripheral blood [35,36]. To investigate whether MMP-9 is involved in sIL-2R release in COVID-19 with lung inflammation, we investigated the associations of sIL-2R levels with plasma MMP-9 and pathological marker levels in CP. We observed that sIL-2R levels positively correlated with MMP-9 levels and negatively correlated with lymphocyte counts and S/F ratio in CP, whereas the correlations between them were not shown in CV (Figure 4B, Table S2).

### 2.5. High Accuracy of Plasma N-Cleaved-Gal9 for Discriminating COVID-19 with Mild Symptoms from COVID-19 with Pneumonia

**Figure 5:**
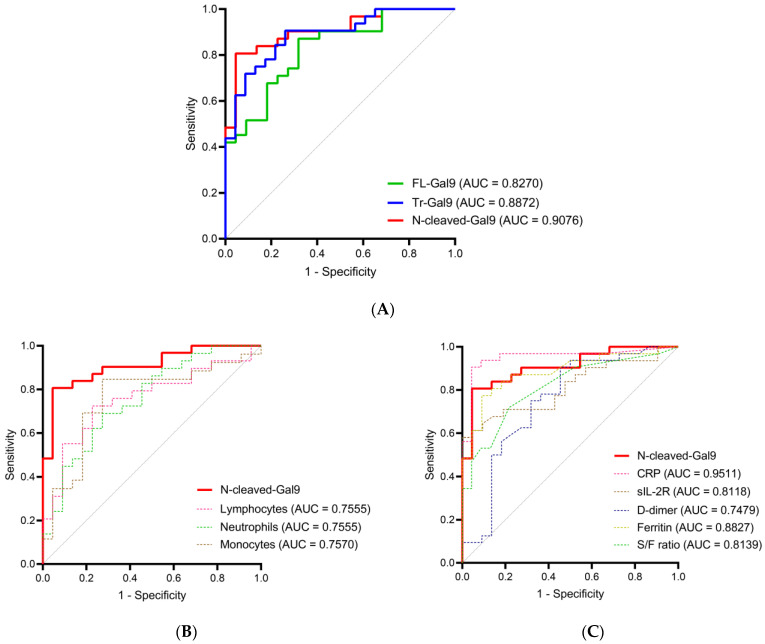
Receiver operating characteristics (ROC) curve for discriminating COVID-19 with mild symptoms from that with pneumonia (**A**–**C**). ROC curve and area under the curve (AUC) value for discriminating CV from CP of plasma N-cleaved-Gal9 were compared with those of (**A**) plasma FL-Gal9 and Tr-Gal9, and (**B**,**C**) specific pathological markers.

To investigate the potential of plasma N-cleaved-Gal9 as a severity marker for COVID-19, we performed a receiver operating characteristic (ROC) curve analysis and compared the area under the curve (AUC) values of plasma Tr-Gal9 and N-cleaved-Gal9 levels for discriminating CV from CP. N-cleaved-Gal-9 levels discriminated CV from CP with high accuracy (AUC: 0.9076) (Figure 5A, Table S3). On the other hand, Tr-Gal9 levels had moderate accuracy (AUC: 0.8872) in discriminating them. The most efficient marker for discriminating them was CRP, followed by N-cleaved-Gal9, Tr-Gal9, ferritin, FL-Gal9, and S/F ratio (Figure 5B,C, Table S3).

### 2.6. Time Course of Plasma Gal-9, Plasma MMPs, and Specific Pathological Markers during TCZ Treatment

**Figure 6:**
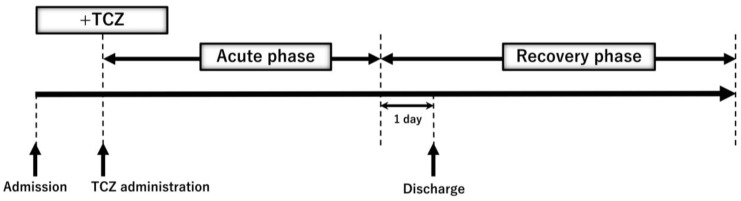
Definition of +TCZ, acute phase, and recovery phase during TCZ treatment in the study. +TCZ was defined as the period immediately before TCZ administration. The recovery phase was defined as the period from the day after TCZ administration to one day before discharge. The recovery phase was defined as the subsequent period.

CP patients with severe symptoms managed in an intensive care unit (ICU) were treated with TCZ (TCZ (+)) and the other CP patients were not treated with TCZ (TCZ (−)) (Figure 2). Pathological marker levels in both groups at their initial visits are shown in Table S4. In TCZ (+) but not TCZ (−) patients, lymphopenia occurred. CRP, sIL-2R, and ferritin levels in both groups and D-dimer levels in TCZ (+) patients were aberrant values. CRP levels in TCZ (+) patients were more than 3.5 mg/dL, which is its cut-off for the beneficial effects of TCZ treatment [37]. The respiratory functions in TCZ (+) patients were significantly exacerbated compared to those in TCZ (−) according to their S/F ratio. In this study, we defined the three phases; (1) the period immediately before TCZ administration, (2) the acute phase, and (3) the recovery phase during TCZ treatment (Figure 6). The acute phase was the period from the day after TCZ administration to one day before discharge, and the recovery phase was the subsequent period.

**Figure 7:**
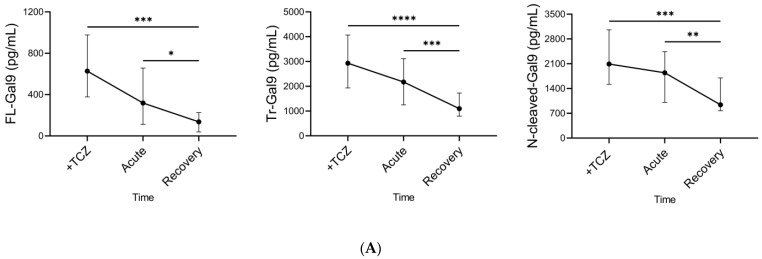

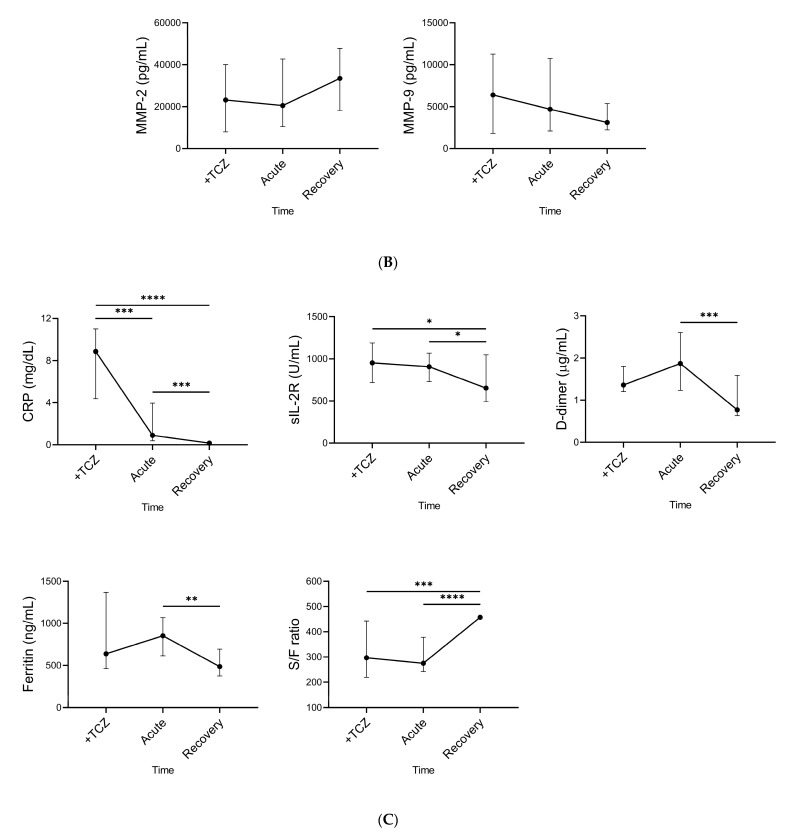
Time course of Gal-9, MMPs, and specific pathological markers during TCZ treatment (**A**–**C**). Changes of (**A**) plasma Gal-9, (**B**) plasma MMP-2 and MMP-9, and (**C**) specific pathological marker levels during TCZ treatments are shown. The median for each parameter in each phase is plotted with IQR. * *p* < 0.05, ** *p* < 0.01, *** *p* < 0.001, **** *p* < 0.0001.

We determined the time course of plasma Gal-9, plasma MMPs, and the specific pathological marker levels during TCZ treatment. Plasma FL-Gal9, Tr-Gal9, and N-cleaved-Gal9 levels significantly decreased after TCZ administration (Figure 7A). MMP-2 and MMP-9 levels were on the rise and decline, respectively (Figure 7B). CRP, sIL-2R, D-dimer, and ferritin levels and S/F ratio were significantly improved (Figure 7C).

### 2.7. Associations between Plasma FL-Gal9 and N-Cleaved-Gal9 during TCZ Treatment

We evaluated the associations between plasma FL-Gal9 and N-cleaved-Gal9 levels during TCZ treatment. There was a positive correlation between FL-Gal9 and N-cleaved-Gal9 levels in the period before TCZ administration, in contrast to no correlation between them in the recovery phase (Figure S2). These results suggested that FL-Gal9 could be cleaved almost as soon as it was released into the peripheral blood in severe conditions of COVID-19.

### 2.8. Associations of Plasma N-Cleaved-Gal9 with sIL-2R during TCZ Treatment

**Figure 8:**
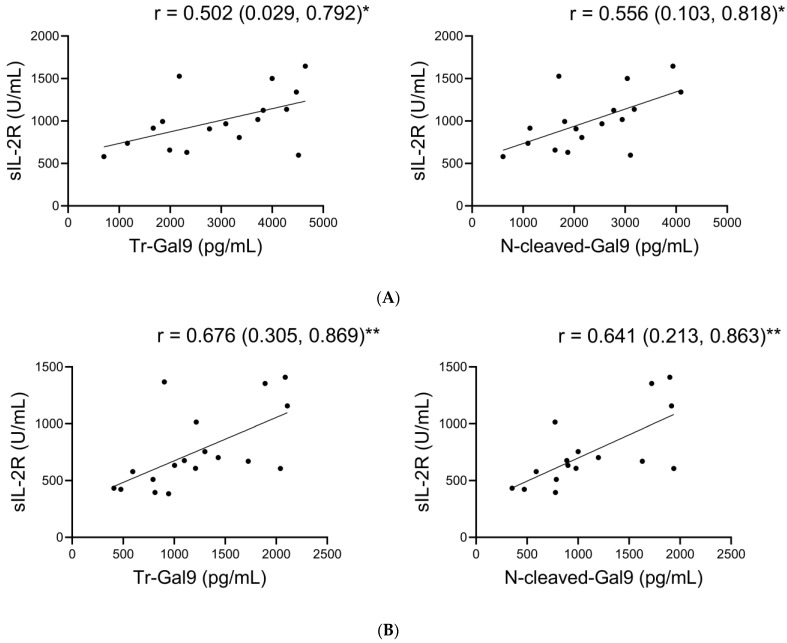
Correlations of Tr-Gal9 and N-cleaved-Gal9 with sIL-2R during TCZ treatments (**A**,**B**). Scatter plots show Spearman’s rank correlations of plasma Tr-Gal9 and N-cleaved-Gal9 levels with sIL-2R levels in (**A**) the period before TCZ treatments and (**B**) the recovery phase. r: correlation coefficient, ( ): 95% CI, * *p* < 0.05, ** *p* < 0.01.

We analyzed the associations of plasma N-cleaved-Gal9 levels with plasma MMPs and the specific pathological marker levels during TCZ treatment. As shown in Figure 8 and Table S5, we observed the positive correlations between N-cleaved-Gal9 and sIL-2R levels as well as between Tr-Gal9 and sIL-2R levels in both the period immediately before TCZ administration and the recovery phase. The correlation coefficients of N-cleaved-Gal9 and Tr-Gal9 levels with sIL-2R levels were nearly equivalent to each other in each phase. FL-Gal-9 levels showed a correlation with sIL-2R levels only in the recovery phase.

### 2.9. Moderate Accuracy of Plasma N-Cleaved-Gal9 for Discriminating the Period before TCZ Administration from the Recovery Phase

**Figure 9:**
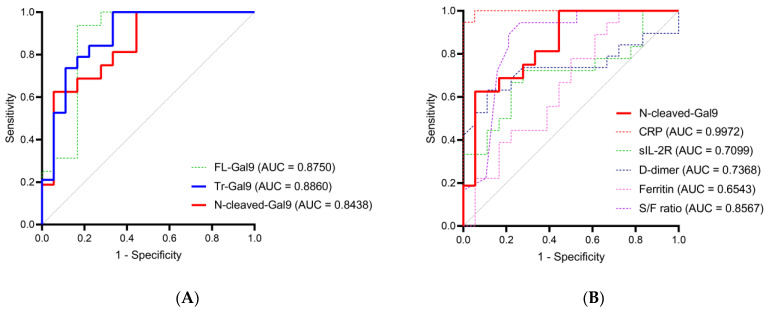
ROC curve for discriminating the period before TCZ administrations from the recovery phase (**A**,**B**). ROC curve and AUC value for discriminating the period immediately before TCZ administration from the recovery phase of plasma N-cleaved-Gal9 were compared with those of (**A**) plasma FL-Gal9 and Tr-Gal9 and (**B**) specific pathological markers.

Finally, we performed a ROC curve analysis to evaluate the utility of plasma N-cleaved-Gal9 for monitoring the therapeutic effects of TCZ. Plasma N-cleaved-Gal-9 levels had moderate accuracy in discriminating the period before TCZ administration from the recovery phase, with its AUC value (AUC: 0.8438) nearly equivalent to that of plasma FL-Gal9 (AUC: 0.8750) and Tr-Gal9 (AUC: 0.8860) levels (Figure 9A, Table S6). As compared to the specific pathological markers, plasma N-cleaved-Gal-9 was the most efficient marker next to CRP (AUC: 0.9972) and S/F ratio (AUC: 0.8567) (Figure 9B).

## 3. Discussion

Our study is the first to investigate the roles of circulating cleaved forms of Gal-9 as a biomarker in COVID-19. We demonstrated that plasma N-cleaved-Gal9 levels increased in COVID-19, and were higher in severe cases with pneumonia compared to mild cases. An increase in plasma N-cleaved-Gal9 levels was predominant in severe COVID-19 rather than severe bacterial infections. We also revealed that plasma N-cleaved-Gal9 were associated with COVID-19 severity and could be a more useful marker than plasma Tr-Gal9 for accurately discriminating the patients associated with pneumonia from those with moderate symptoms among COVID-19. Furthermore, we found that plasma N-cleaved-Gal9 levels gradually decreased and were associated with inflammatory marker levels throughout TCZ treatment. Our ROC curve analysis indicated that plasma N-cleaved-Gal9 had sufficient accuracy in reflecting the therapeutic effects of TCZ.

Two factors could mutually affect a marked increase of plasma N-cleaved-Gal9 levels in COVID-19 with pneumonia. One is the promotion of FL-Gal9 release in COVID-19 with pneumonia compared to the other groups. FL-Gal9 is susceptible to cleavage within a linker peptide; thus, the higher amount of plasma FL-Gal9 can explain an increase in plasma N-cleaved-Gal9. Another is an enhancement of FL-Gal9 being processed by proteases shortly after FL-Gal9 is released into the blood in COVID-19 with pneumonia. Clarifying the mechanisms that cause an increase in circulating cleaved Gal-9 in severe COVID-19 is essential in the future.

We illustrated that plasma N-cleaved-Gal9 levels have a high sensitivity in reflecting the severity of COVID-19, primarily being associated with inflammatory levels. Plasma Gal-9 shows positive correlations with proinflammatory mediators, especially IL-6, IP-10, and CRP, and a negative correlation with an anti-inflammatory cytokine, transforming growth factor-β (TGF-β), in COVID-19 patients [15]. Moreover, exogenous Gal-9 diminishes SARS-CoV-2 infection and replication in a mouse model [38]. Mice treated with Gal-9 exhibit increased survival rates potentiating cytokine and chemokine productions in bronchoalveolar lavage fluid and inducing effector T cell and DC responses in lung tissues. Our clinical findings on N-cleaved-Gal9 in COVID-19 may provide insight into the relevance of Gal-9 proteolysis to its function of inducing antiviral immune responses against SARS-CoV-2 infection.

It is well-known that peripheral D-dimer, an important coagulation marker for evaluating thrombosis, significantly increases in COVID-19, and its levels are associated with the disease severity [39,40,41,42,43]. On the other hand, Japanese COVID-19 patients requiring oxygen or mechanical ventilation show normal to abnormal levels of D-dimer, although these patients include more cases with abnormal levels than mild COVID-19 patients [44]. In our study, plasma D-dimer levels seemed to increase more in the cases with pneumonia than in the mild cases; however, there was no statistical difference between the two groups. The reason for the lack of statistical significance could be explained by both the wide range of D-dimer levels in the patients with pneumonia and the small sample size in our study.

We observed increased levels of plasma MMP-2 and comparable levels of plasma MMP-9 in COVID-19 with pneumonia compared to healthy controls. By contrast, a previous study has indicated a decrease in plasma MMP-2 and an increase in plasma MMP-9 in COVID-19 [28]. Another study has also reported higher levels of serum MMP-9 in severe COVID-19 [29]. The difference in the results of our study and the previous study [28] might be explained by the different methods used for the measurements of MMP-2 and MMP-9. The previous study used a gelatin zymography, while we used an immunoassay. MMP-9 is involved in acute lung injury and ARDS, degrading the alveolar-capillary barrier. In contrast, it has a pivotal role in alveolar epithelial repair [45]. We showed the associations of serum sIL-2R with plasma MMP-9 and lymphocyte counts in COVID-19 with pneumonia. sIL-2R processed by MMP-9 in inflamed lung tissues and/or on infiltrated lymphocytes, as observed in other diseases [35,36], might be released in COVID-19 with pneumonia in our study. We additionally showed the associations of plasma N-cleaved-Gal9 with plasma MMP-9 and serum sIL-2R in these patients, suggesting that MMP-9 may be involved in the cleavage of Gal-9 as well as sIL-2R.

We demonstrated that plasma N-cleaved-Gal9 levels decreased when inflammation, coagulopathy, and respiratory failure levels were reduced after TCZ administrations. A decrease in plasma N-cleaved-Gal9 was associated with the suppression of inflammatory responses during TCZ treatment. Of note, there is also the possibility that inflammation is still present even after the recovery phase because COVID-19 survivors often develop pulmonary fibrosis characterized by chronic inflammation [46,47]. The correlation of N-cleaved-Gal9 levels with sIL-2R levels in the recovery phase may indicate an involvement of cleaved-Gal-9 in persistent inflammation. Gal-9 can be implicated in the process of post-ARDS lung fibrosis in COVID-19 [48]. However, we observed that the patients did not present pulmonary fibrosis after TCZ treatment in this study.

As demonstrated in ROC curve analysis, plasma N-cleaved-Gal9 had sufficient accuracy in reflecting the therapeutic effects of TCZ in severe COVID-19. Meanwhile, CRP levels showed more prominent sensitivity and specificity when compared to plasma N-cleaved-Gal9. CRP levels is directly reduced by the influence of TCZ because TCZ inhibits IL-6 signaling that contributes to the synthesis of CRP [49]. In our study, dexamethasone was administered in combination with TCZ. Combined dexamethasone and TCZ treatment also suppress CRP levels [50]. Thus, a decline in CRP levels does not directly reflect the reduction of inflammatory levels by TCZ. We confirmed that N-cleaved-Gal9 could be a surrogate clinical marker for evaluating the therapeutic effects of TCZ.

Some limitations exist in this study. Elucidating the circulating levels of cleaved Gal-9 in illness did not allow us to directly assess the occurrence of Gal-9 proteolysis in the pathogenesis. We need to clarify whether or not Gal-9 directly interacts with several proteases, such as MMPs, in an in vitro model of SARS-CoV-2 infection. We suggested that Gal-9 could be cleaved as soon as its release into blood in severe conditions but not the recovery phase by TCZ treatment, implying that inhibition of the IL-6 signaling cascade in severe COVID-19 may enhance the subsidence of Gal-9 proteolysis. In the future, it is essential to investigate how the interactions of Gal-9 with proteases are significant in both the hyperinflammatory and immunosuppressive responses developing severe COVID-19. Here, we had a small number of healthy controls and COVID-19 patients. We need a larger-scale study to find the precise outcomes of plasma N-cleaved-Gal9 in COVID-19 and accurately assess the availability of plasma N-cleaved-Gal9 as a marker for determining COVID-19 severity and therapeutic effects of TCZ.

## 4. Materials and Methods

### 4.1. Study Design and Participants

This study was a cross-sectional analytical study. Of 105 patients with fever recruited in Sendai City Hospital (SCH), Sendai, Japan, 62 patients in 2020 were included in our previous study [23], and 43 patients in 2021 were enrolled in our study (Figure 2). We sampled the patients’ EDTA plasma obtained by centrifugation of the peripheral blood at 3000 rpm for 10 min. Among them, 55 patients were diagnosed with COVID-19 by the PCR test for SARS-CoV-2 of a nasopharyngeal sample as previously described [26]. It was assumed that COVID-19 patients in 2020 were mostly infected with the B.1.1.284 variant of SARS-CoV-2, and those in 2021 were infected with the B.1.1.214 variant, followed by the R.1. variant [51]. The remaining PCR-negative 50 patients were clinically diagnosed with bacterial infection (ID). We divided the 55 patients infected with SARS-CoV-2 into two groups: 23 patients with mild clinical symptoms (CV) and 32 patients associated with pneumonia (CP) according to clinical diagnosis based on the symptoms, chest computed tomography (CT), and SpO2 and FiO2 tests. The severity of symptoms in each patient was assessed with reference to the WHO classification as described [52]. Of CP patients, those with severe symptoms being managed in ICU were treated with TCZ in combination with dexamethasone (TCZ (+)). The measurements of plasma FL-Gal9, Tr-Gal9, MMP-2, and MMP-9 and laboratory tests for the specific pathological markers were performed in CV, CP, ID, and TCZ (+) patients. The plasma of 30 healthy controls that were negative for HIV, SARS-CoV-2, and hepatitis B and C viruses were obtained from BioIVT (Hicksville, NY, USA) and subjected to the measurements of plasma FL-Gal9, Tr-Gal9, MMP-2, and MMP-9.

### 4.2. Determination of Plasma FL-Gal9, Tr-Gal9, and N-Cleaved-Gal9

The plasma levels of FL-Gal9 were quantified using FL-Gal-9 ELISA (GalPharma, Takamatsu, Japan), described previously [53]. In this ELISA, 9S2-3 monoclonal antibody (Ab) (GalPharma, Takamatsu, Japan) against NCRD of human Gal-9 and polyclonal Ab (GalPharma, Takamatsu, Japan) against its CCRD were used as the capture and detection antibodies, respectively (Figure 1). The plasma levels of Tr-Gal9 were quantified using Tr-Gal-9 ELISA, which has been previously constructed using both the capture monoclonal Ab, 9S2-3, and detection monoclonal Ab, biotinylated ECA8 (MBL, Nagoya, Japan), against NCRD of human Gal-9 [54]. The N-cleaved-Gal9 levels were calculated by subtracting the concentration measured by FL-Gal9 ELISA from that measured by Tr-Gal9 ELISA.

### 4.3. Measurements of Plasma MMP-2 and -9

Plasma MMP-2 and MMP-9 levels were measured using a Milliplex MAP assay kit (Millipore, Burlington, MA, USA, cat#HMMP2MAG-55K) according to the manufacturer’s protocol. The data were acquired using a Bio-Plex 200 (Bio-Rad, Hercules, CA, USA) instrument and analyzed using Bio-Plex manager software (Bio-Rad).

### 4.4. Measurements of Specific Pathological Markers That Reflect COVID-19 Severity

The data of SpO2/FiO2 ratio, chest CT, and conventional laboratory findings, including the counts of WBC, PLT, RBC, lymphocyte, neutrophils, and monocyte, the serum levels of CRP, sIL-2R, ferritin, and the plasma levels of D-dimer were obtained through Sendai City Hospital as described [26].

### 4.5. Statistical Analyses

Statistical analyses were performed using GraphPad Prism 8 (GraphPad Software, San Diego, CA, USA) and R statistical software (ver. 4.2.0; The R Foundation for Statistical Computing, Vienna, Austria). The levels of each parameter were expressed as the median with IQR. Mann–Whitney U test and Kruskal–Wallis test were used to assess the differences between two groups and multiple groups, respectively. Correlations were assessed by Spearman’s rank correlation analysis. *p* values less than 0.05 were considered statistically significant.

## 5. Conclusions

Increased levels of plasma N-cleaved-Gal9 were associated with the severity of COVID-19. MMP-9 may involve the generation of N-cleaved-Gal9 and sIL-2R. A decrease in plasma N-cleaved-Gal9 levels was associated with the suppression of inflammatory responses during TCZ treatment. We propose that plasma N-cleaved-Gal9 could be a surrogate marker for determining the severity of COVID-19 and monitoring the therapeutic effects of TCZ. Our results shed new light on the relevance of proteolytic products of Gal-9 in the pathophysiology and clinical management of COVID-19.

## Figures and Tables

**Table 1:** Characteristics of the patients enrolled in the study.

	Reference Range ^§^	CV	CP	ID	*p*-Value
n		23	32	50	
Median Age (Range)		22 (19–102)	53 (20–99)	80 (23–94)	<0.0001
Male (n)		13	27	34	-
Female (n)		10	5	16	-
WBC (10^3^/μL)	M 3.6–9.0 F 3.0–7.8	4.9 (3.9–6.1)	5.5 (4.5–6.7)	10.3 (7.8–12.4)	<0.0001
PLT (10^4^/μL)	13.8–30.9	23.0 (21.2–27.0)	21.6 (15.5–36.2)	23.5 (17.9–28.2)	0.9134
RBC (10^6^/μL)	M 3.87–5.25 F 3.53–4.66	5.28 (4.57–5.46)	4.71 (4.34–5.01)	4.04 (3.36–4.55)	<0.0001
Comorbidity					
Hypertension		0 (0.00%)	12 (37.50%)	5 (10.00%)	
Diabetes		1 (4.35%)	10 (31.25%)	5 (10.00%)	
Cancer		0 (0.00%)	5 (15.63%)	11 (22.00%)	
Hyperlipidemia		0 (0.00%)	4 (12.50%)	3 (6.00%)	
Allergy		1 (4.35%)	4 (12.50%)	1 (2.00%)	
Cardiovascular diseases		0 (0.00%)	3 (9.38%)	6 (12.00%)	
Kidney diseases		0 (0.00%)	3 (9.38%)	1 (2.00%)	
Cerebrovascular diseases		0 (0.00%)	2 (6.25%)	7 (14.00%)	
Others		7 (30.43%)	20 (40.00%)	20 (40.00%)	

The median for each hematological parameter is indicated with the interquartile range (IQR). The case number of each comorbidity is indicated with its percentage to the total comorbidity. M: male, F: female, WBC: white blood cell, PLT: platelet, RBC: red blood cell, ^§^ Reference ranges in Japan.

## Data Availability

Not applicable.

## Supplementary Materials

The following supporting information can be downloaded at: https://www.mdpi.com/article/10.3390/ijms24043591/s1.
