# Supplementary material for: Plasma N-Cleaved Galectin-9 Is a Surrogate Marker for Determining the Severity of COVID-19 and Monitoring the Therapeutic Effects of Tocilizumab
Source: Int J Mol Sci. 2023 Feb 10;24(4):3591. doi: 10.3390/ijms24043591 (PMC9964849; doi:10.3390/ijms24043591)
Supplement: Supplementary file 1 [file ijms-24-03591-s001.zip › Figure S1.pdf]

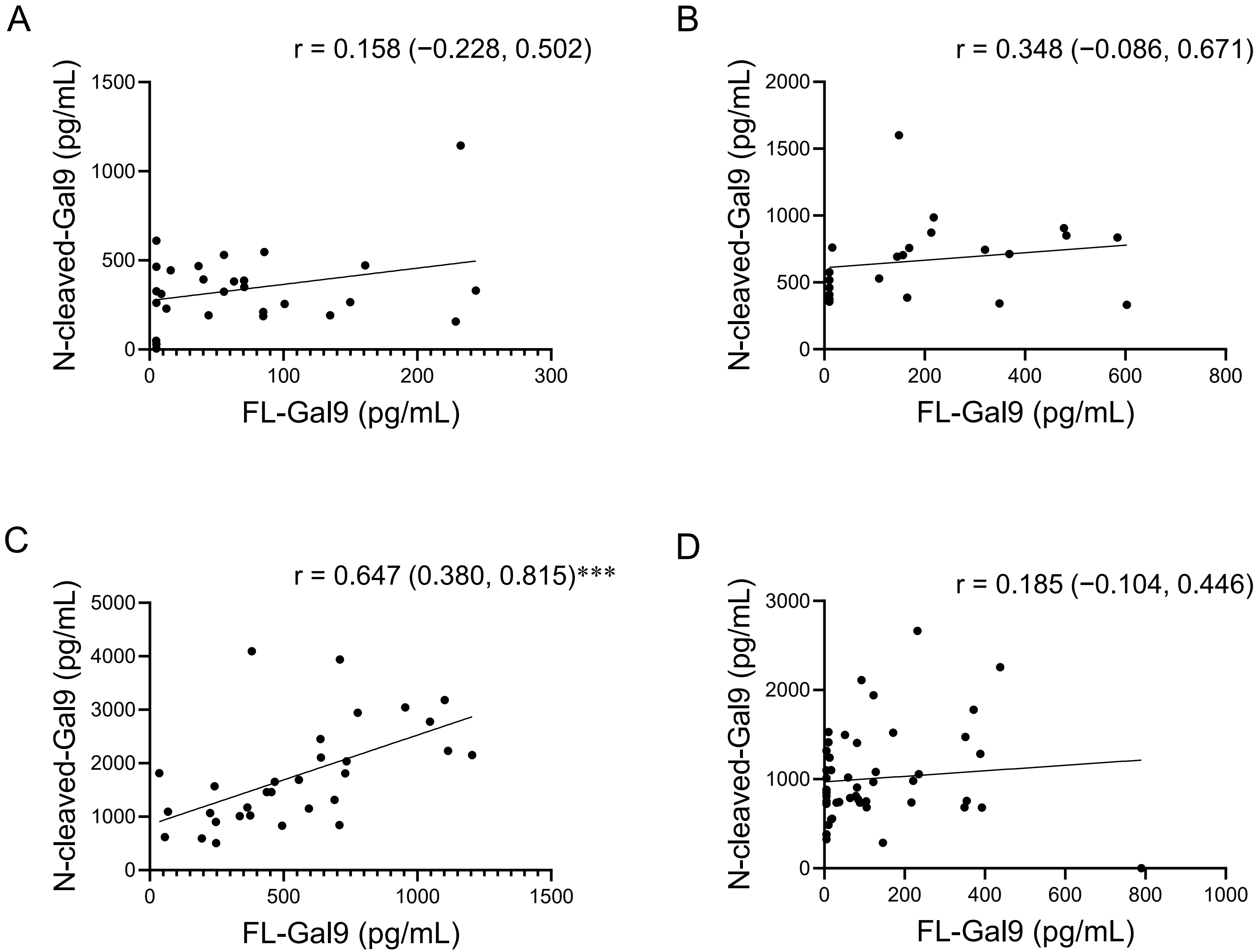

Figure S1. Correlations between plasma FL-Gal9 and N-cleaved-Gal9 levels in healthy controls, CV, CP, and ID (A-D). Scatter plots show Spearman's rank correlations between FL-Gal9 and N-cleaved-Gal9 levels in A) healthy controls, B) CV, C) CP, and D) ID. r: correlation coefficient, (): 95% confidence interval. ***p<0.001.
