# Supplementary material for: Plasma N-Cleaved Galectin-9 Is a Surrogate Marker for Determining the Severity of COVID-19 and Monitoring the Therapeutic Effects of Tocilizumab
Source: Int J Mol Sci. 2023 Feb 10;24(4):3591. doi: 10.3390/ijms24043591 (PMC9964849; doi:10.3390/ijms24043591)
Supplement: Supplementary file 1 [file ijms-24-03591-s001.zip › Figure S2.pdf]

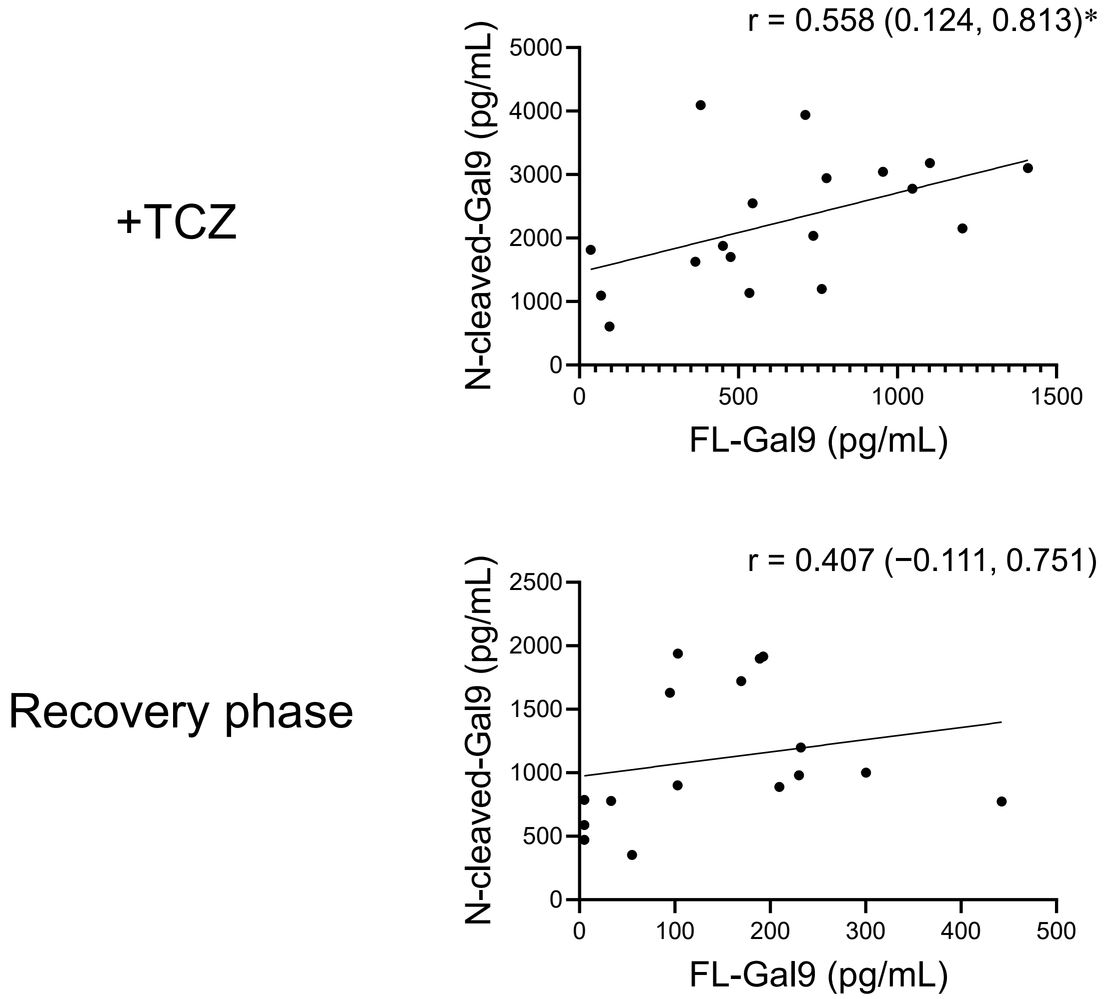

Figure S2. Correlations between plasma FL-Gal9 and N-cleaved-Gal9 levels during TCZ treatment. Scatter plots show Spearman's rank correlations between FL-Gal9 and N-cleaved-Gal9 levels in the period immediately before TCZ (+TCZ) and recovery phase. r: correlation coefficient, (): 95% confidence interval. *p<0.05
