# Supplementary material for: Plasma N-Cleaved Galectin-9 Is a Surrogate Marker for Determining the Severity of COVID-19 and Monitoring the Therapeutic Effects of Tocilizumab
Source: Int J Mol Sci. 2023 Feb 10;24(4):3591. doi: 10.3390/ijms24043591 (PMC9964849; doi:10.3390/ijms24043591)
Supplement: Supplementary file 1 [file ijms-24-03591-s001.zip › Table S1.pdf]

Table S1. Spearman's rank correlations of plasma Gal9 levels with plasma MMPs and specific pathological marker levels in CP, CV, and ID.

		CP			CV			ID		
		**r**	**95%CI**	***p***	**r**	**95%CI**	***p***	**r**	**95%CI**	***p***
**FL-Gal9**	**MMP-2**	-0.204	-0.521, 0.162	0.270	0.228	-0.226, 0.600	0.321	0.296	0.003, 0.543	<0.05
	**MMP-9**	0.397	0.042, 0.662	<0.05	-0.068	-0.476, 0.364	0.762	0.421	0.112, 0.655	<0.05
	**Lymphocytes**	-0.425	-0.689, -0.062	<0.05	0.376	-0.067, 0.695	0.093	ND		
	**Neutrophils**	0.120	-0.265, 0.472	0.540	-0.198	-0.580, 0.255	0.389	ND		
	**Monocytes**	0.047	-0.347, 0.426	0.822	0.022	-0.414, 0.449	0.926	ND		
	**CRP**	0.405	0.059, 0.664	<0.05	0.126	-0.313, 0.520	0.578	0.088	-0.201, 0.363	0.553
	**sIL-2R**	0.522	0.200, 0.743	<0.01	0.286	-0.166, 0.639	0.208	ND		
	**D-dimer**	0.523	0.206, 0.740	<0.01	0.194	-0.259, 0.578	0.3984	0.106	-0.184, 0.379	0.474
	**Ferritin**	0.460	0.120, 0.704	<0.05	0.369	-0.075, 0.691	0.100	0.012	-0.309, 0.331	0.942
	**S/F ratio**	-0.310	-0.599, 0.050	0.090	0.337	-0.098, 0.664	0.125	-0.127	-0.405, 0.173	0.407
**Tr-Gal9**	**MMP-2**	-0.129	-0.457, 0.230	0.479	0.096	-0.350, 0.507	0.678	0.023	-0.266, 0.308	0.880
	**MMP-9**	0.472	0.142, 0.708	<0.01	-0.030	-0.446, 0.397	0.896	0.337	0.014, 0.596	<0.05
	**Lymphocytes**	-0.551	-0.763, -0.231	<0.01	0.273	-0.168, 0.623	0.219	ND		
	**Neutrophils**	0.274	-0.103, 0.582	0.150	-0.265	-0.617, 0.176	0.233	ND		
	**Monocytes**	0.284	-0.116, 0.605	0.160	0.053	-0.377, 0.464	0.817	ND		
	**CRP**	0.519	0.208, 0.735	<0.01	0.320	-0.106, 0.647	0.137	0.137	-0.147, 0.400	0.343
	**sIL-2R**	0.657	0.394, 0.820	<0.0001	0.266	-0.187, 0.626	0.242	ND		
	**D-dimer**	0.650	0.390, 0.814	<0.0001	0.201	-0.241, 0.574	0.370	0.221	-0.061, 0.471	0.122

	**Ferritin**	0.579	0.283, 0.774	<0.001	0.206	-0.236, 0.578	0.357	0.126	-0.193, 0.421	0.437
	**S/F ratio**	-0.540	-0.748, -0.236	<0.01	0.405	-0.009, 0.700	0.056	-0.078	-0.358, 0.214	0.600
**N-cleaved-Gal9**	**MMP-2**	-0.252	-0.557, 0.112	0.171	-0.003	-0.434, 0.430	0.991	-0.066	-0.347, 0.225	0.658
	**MMP-9**	0.519	0.195, 0.741	<0.01	-0.014	-0.433, 0.410	0.952	0.200	-0.132, 0.492	0.234
	**Lymphocytes**	-0.527	-0.752, 0.191	<0.01	0.078	-0.366, 0.493	0.737	ND		
	**Neutrophils**	0.305	-0.076, 0.609	0.114	-0.214	-0.591, 0.240	0.349	ND		
	**Monocytes**	0.381	-0.008, 0.669	0.055	0.256	-0.198, 0.619	0.262	ND		
	**CRP**	0.570	0.270, 0.769	<0.01	0.465	0.054, 0.741	<0.05	0.147	-0.137, 0.409	0.308
	**sIL-2R**	0.789	0.598, 0.895	<0.0001	0.181	-0.272, 0.568	0.432	ND		
	**D-dimer**	0.656	0.392, 0.820	<0.0001	-0.039	-0.463, 0.399	0.867	0.208	-0.075, 0.460	0.148
	**Ferritin**	0.577	0.273, 0.776	<0.01	-0.058	-0.478, 0.383	0.801	0.133	-0.186, 0.427	0.413
	**S/F ratio**	-0.608	-0.792, -0.323	<0.001	0.363	-0.069, 0.681	0.097	-0.052	-0.334, 0.239	0.729

r: correlation coefficient, CI: confidence interval, ND: no data.
