# Supplementary material for: Plasma N-Cleaved Galectin-9 Is a Surrogate Marker for Determining the Severity of COVID-19 and Monitoring the Therapeutic Effects of Tocilizumab
Source: Int J Mol Sci. 2023 Feb 10;24(4):3591. doi: 10.3390/ijms24043591 (PMC9964849; doi:10.3390/ijms24043591)
Supplement: Supplementary file 1 [file ijms-24-03591-s001.zip › Table S2.pdf]

Table S2. Spearman's rank correlations of sIL-2R levels with plasma MMP-9 and specific pathological marker levels in CP, CV, and ID.

		**CP**			**CV**			**ID**		
		**r**	**95%CI**	***p***	**r**	**95%CI**	***p***	**r**	**95%CI**	***p***
**sIL-2R**	**MMP-9**	0.441	0.096, 0.691	<0.05	-0.505	-0.769, -0.094	<0.05	ND		
	**Lymphocytes**	-0.484	-0.723, -0.143	<0.01	-0.191	-0.575, 0.262	0.405	ND		
	**CRP**	0.732	0.510, 0.863	<0.0001	0.193	-0.261, 0.576	0.403	ND		
	**D-dimer**	0.760	0.556, 0.878	<0.0001	0.250	-0.216, 0.624	0.288	ND		
	**Ferritin**	0.504	0.183, 0.728	<0.01	0.240	-0.214, 0.608	0.295	ND		
	**S/F ratio**	-0.637	-0.809, -0.365	<0.001	-0.277	-0.633, 0.175	0.224	ND		
