# Supplementary material for: Plasma N-Cleaved Galectin-9 Is a Surrogate Marker for Determining the Severity of COVID-19 and Monitoring the Therapeutic Effects of Tocilizumab
Source: Int J Mol Sci. 2023 Feb 10;24(4):3591. doi: 10.3390/ijms24043591 (PMC9964849; doi:10.3390/ijms24043591)
Supplement: Supplementary file 1 [file ijms-24-03591-s001.zip › Table S3.pdf]

Table S3. Accuracies of plasma Gal-9 and specific pathological marker levels for discriminating CV from CP.

	AUC	Standard error	Cut off	Youden Index	Sensitivity	Specificity
**FL-Gal9**	0.8270	0.05597	221.6	0.5528	0.871	0.6818
**Tr-Gal9**	0.8872	0.04363	1117	0.6454	0.9063	0.7391
**N-cleaved-Gal9**	0.9076	0.04050	998.7	0.761	0.8065	0.9545
**Lymphocytes**	0.7555	0.06876	1246	0.4968	0.7241	0.7727
**Neutrophils**	0.7555	0.06834	2990	0.417	0.6897	0.7273
**Monocytes**	0.7570	0.07340	391.7	0.5735	0.8462	0.7273
**CRP**	0.9511	0.03159	0.5300	0.8628	0.9063	0.9565
**sIL-2R**	0.8118	0.05883	721.0	0.5806	0.5806	1.0000
**D-dimer**	0.7479	0.07243	0.5700	0.4518	0.9063	0.5455
**Ferritin**	0.8827	0.04669	292.5	0.6833	0.7742	0.9091
**S/F ratio**	0.8139	0.05730	459.5	0.5014	0.7188	0.7826
