# Supplementary material for: Plasma N-Cleaved Galectin-9 Is a Surrogate Marker for Determining the Severity of COVID-19 and Monitoring the Therapeutic Effects of Tocilizumab
Source: Int J Mol Sci. 2023 Feb 10;24(4):3591. doi: 10.3390/ijms24043591 (PMC9964849; doi:10.3390/ijms24043591)
Supplement: Supplementary file 1 [file ijms-24-03591-s001.zip › Table S4.pdf]

Table S4. Laboratory findings of CP patients treated with and without TCZ at their initial visits.

	**TCZ (-)**	**TCZ (+)**	***p* value**
**n**	13	19	-
**Lymphocytes (/μL)**	1226 (944.3-1689)	836.0 (532.5-1143)	0.0647
**Neutrophils (/μL)**	3279 (2418-4318)	4720 (3038-5830)	0.0594
**Monocytes (/μL)**	480.0 (408.0-569.6)	495.0 (418.0-660.0)	0.4823
**CRP (mg/dL)**	1.940 (1.010-4.740)	8.870 (4.380-11.00)	<0.01
**sIL-2R (U/mL)**	725.5 (518.0-969.0)	953.0 (718.0-1188)	0.0699
**D-dimer (μg/mL)**	0.9200 (0.6850-1.160)	1.360 (1.200-1.805)	<0.01
**Ferritin (ng/mL)**	357.0 (219.3-711.8)	638.0 (460.0-1368)	0.0531
**S/F ratio**	457.1 (442.9-464.3)	321.4 (219.5-457.1)	<0.01

TCZ (+): CP patients treated with TCZ, TCZ (-): CP patients not treated with TCZ, the median value for each parameter was indicated with IQR, (): IQR.
