# Supplementary material for: Plasma N-Cleaved Galectin-9 Is a Surrogate Marker for Determining the Severity of COVID-19 and Monitoring the Therapeutic Effects of Tocilizumab
Source: Int J Mol Sci. 2023 Feb 10;24(4):3591. doi: 10.3390/ijms24043591 (PMC9964849; doi:10.3390/ijms24043591)
Supplement: Supplementary file 1 [file ijms-24-03591-s001.zip › Table S5.pdf]

Table S5. Spearman's rank correlations of plasma Gal-9 levels with sIL-2R levels in the patients during TCZ treatment.

		Phase	r	95% CI	*p*
sIL-2R	FL-Gal9	+TCZ	0.181	-0.328, 0.609	0.485
		Recovery	0.723	0.354, 0.897	<0.01
	Tr-Gal9	+TCZ	0.502	0.029, 0.792	<0.05
		Recovery	0.676	0.305, 0.869	<0.01
	N-cleaved-Gal9	+TCZ	0.556	0.103, 0.818	<0.05
		Recovery	0.641	0.213, 0.863	<0.01

r: correlation coefficient, CI: confidence interval.
