# Supplementary material for: Plasma N-Cleaved Galectin-9 Is a Surrogate Marker for Determining the Severity of COVID-19 and Monitoring the Therapeutic Effects of Tocilizumab
Source: Int J Mol Sci. 2023 Feb 10;24(4):3591. doi: 10.3390/ijms24043591 (PMC9964849; doi:10.3390/ijms24043591)
Supplement: Supplementary file 1 [file ijms-24-03591-s001.zip › Table S6.pdf]

Table S6. Accuracies of plasma Gal-9 and specific pathological marker levels for discriminating the period immediately before TCZ administration from the recovery phase.

	AUC	Standard error	Cut off	Youden Index	Sensitivity	Specificity
**FL-Gal9**	0.8750	0.06595	332.5	0.7708	0.9375	0.8333
**Tr-Gal9**	0.8860	0.05633	2143	0.6667	1.000	0.6667
**N-cleaved-Gal9**	0.8438	0.06694	1048	0.5694	0.6250	0.9444
**CRP**	0.9972	0.004629	2.100	0.9474	1.000	0.9474
**sIL-2R**	0.7099	0.08912	720.5	0.4445	0.6667	0.7778
**D-dimer**	0.7368	0.08834	1.000	0.5205	0.6316	0.8889
**Ferritin**	0.6543	0.09234	914.0	0.2778	0.8889	0.3889
**S/F ratio**	0.8567	0.06638	428.6	0.6812	0.9444	0.7368
